# Association between a history of depression and anti-müllerian hormone among late-reproductive aged women: the Harvard study of moods and cycles

**DOI:** 10.1186/s40695-020-00056-x

**Published:** 2020-09-01

**Authors:** Samuel W. Golenbock, Lauren A. Wise, Geralyn M. Lambert-Messerlian, Elizabeth E. Eklund, Bernard L. Harlow

**Affiliations:** 1 Department of Epidemiology, Boston University School of Public Health, 715 Albany St, Boston, MA 02118 USA; 2 Department of Pathology and Laboratory Medicine, Alpert Medical School at Brown University, 222 Richmond St, Providence, RI 02903 USA; 3 Women and Infants Hospital, 101 Dudley Street, Providence, RI 02905 USA

**Keywords:** Anti-müllerian hormone, Depression, premenopausal women, Ovarian reserve

## Abstract

**Background:**

There is conflicting evidence regarding the association between a history of depression and risk of early menopause. In a cohort of premenopausal women, we investigated the association between depression history and ovarian reserve, as measured by anti-müllerian hormone (AMH).

**Methods:**

The Harvard Study of Moods and Cycles (HSMC) was a prospective cohort study of women living in the Boston, MA metropolitan-area (1995–1999). Women aged 36–45 years at cohort entry (1995) were sampled from seven Boston metropolitan-area communities using census directories. We measured serum AMH in early-follicular phase venous blood specimens from 141 women with a Structured Clinical Interview for DSM-IV (SCID)-confirmed history of depression and 228 without such a history. We calculated prevalence ratios (PR) for the association between characteristics of depression history and low AMH (≤1.4 ng/mL), adjusting for several potential confounders.

**Results:**

The prevalence of low AMH was similar among depressed (57.5%) and non-depressed (57.9%) women (Adjusted [Adj] PR = 0.90, 95% CI: 0.75, 1.08). Among depressed women, results were not appreciably different among those who had ever used antidepressants and those with comorbid anxiety. Modest inverse associations between depression and low AMH were seen among women aged 36–40 years (Adj PR = 0.75, 95% CI: 0.52, 1.09) and nulliparous women (Adj PR = 0.77, 95% CI: 0.59, 1.00). No dose-response association with greater duration or length of depressive symptoms was observed.

**Conclusions:**

Overall, the prevalence of low AMH was similar for depressed and non-depressed women 36–45 years of age. Surprisingly, among younger and nulliparous women, those with a history of depression had a slightly reduced prevalence of low AMH relative to those without such a history. These results do not indicate reduced ovarian reserve among women with a history of depression.

## Introduction

As women age, they encounter profound changes in reproductive function and associated health. Ovarian reserve is established at birth and declines with age, such that women in menopause have essentially lost their store of ovarian follicles and no longer experience menstrual cycles. The transition to menopause is a complex process involving multiple stages characterized by endocrine changes, dysregulated folliculogenesis, and physiologic and psycho-social symptoms. The menopausal transition has been studied in detail and characterized according to stages in a prospective, longitudinal, multi-center Study of Women’s Health Across the Nation [1]. Changes in mood, sleep, and headache commonly occur as women transition to menopause [2]. In particular, there is evidence that symptoms of depression increase over the course of the menopausal transition [3]. The biological basis for increased risk of depression with aging among women has not been determined with certainty.

Declining endogenous estradiol concentrations are a hallmark of the menopausal transition and may be associated with susceptibility to depression [4]. For example, depressive episodes during the menopausal transition cluster in the late stages coincident with significant loss of estradiol, and other studies reported that depressive symptoms declined with exogenous estradiol treatment [5].

Age-related depression in women and various reproductive hormones have been studied extensively in the past, with the exception of anti-müllerian hormone. Anti-müllerian hormone (AMH) is a glycoprotein hormone produced by the granulosa cells of pre-antral ovarian follicles that acts as a local modulator of folliculogenesis. As women age and the pool of growing ovarian follicles declines, there is a corresponding decrease in serum AMH levels. The decline in circulating AMH occurs throughout the reproductive lifespan until about one year before menopause, when levels become undetectable by current assay methods [6–8]. Prospective studies have found that serum levels of AMH are highly predictive of age at natural menopause [8–11], including those with early onset [10]. Furthermore, because AMH is an intra-ovarian factor produced in the small pre-antral follicles, there is relatively little variation in serum levels across the normal menstrual cycle [12, 13]. It is therefore considered a convenient biomarker as blood specimens can be drawn on any day of the cycle.

The age at which natural menopause occurs varies considerably among women, between 44 and 54 years, with an average age of 51 years [14, 15]. In addition to chronological age, the rate of decline in ovarian reserve is influenced by genetics [16], smoking [6], and perhaps ethnicity [17] among other variables. Smoking, poor self-related health, and high physical activity have been associated with an earlier age at final menstrual period [18]. Some studies suggest that a history of major depression may also be associated with an earlier decline in ovarian function. In one case-control study, Harlow et al. [19] observed an association between a self-reported history of medically-treated depression and early natural menopause. In a later study by the same investigators, women with a history of depression who reported antidepressant use were found to have nearly three times the rate of onset of perimenopause relative to those without a history of depression [20].

The association between depression and AMH concentrations is unexplored to our knowledge. We performed a cross-sectional analysis to test the hypothesis of an association between a history of major depression and ovarian reserve, as measured by AMH, in a cohort of premenopausal women.

We hypothesize that women with a history of depression may show an earlier or more pronounced age-related decrease in serum AMH levels compared to women with no history of major depression.

## Materials and methods

### Study population

Data for the current study were derived from the Harvard Study of Moods and Cycles (HSMC). The HSMC (1995–1999) was a prospective cohort study of late reproductive-aged women with and without a history of major depressive disorder (MDD). The study cohort was recruited from a population-based sample of approximately 6000 women between 36 and 45 years of age residing in seven Boston metropolitan area communities, identified using census directories.

Eligible women were mailed an initial two-page screening questionnaire to assess current menopausal status and depression history. Women completed the 20-item Center for Epidemiologic Studies (CES-D) depression symptoms inventory. CES-D scores of 24 or greater are highly suggestive of major depression, while scores below 16 indicate mild to no depressive symptoms [21]. After receiving a 74% response rate (*N* = 4572), and excluding an additional 408 individuals who did not meet pre-specified inclusion criteria (e.g. outside specified age range, postmenopausal), the target population consisted of 4161 women from which a sample of those with and without a history of MDD could be drawn.

As part of the initial screening, participants with CES-D scores > 24 or a self-reported history of depression that required pharmacological or psychiatric therapy were invited to be enrolled as the cohort with a depression history. Those with CES-D scores < 16 *and* no self-reported history of depression history or treatment were invited to be enrolled as the cohort without a depression history. Eligible women further participated in structured clinical interviews for depression (SCID) based on the *Diagnostic and Statistical Manual of Mental Disorders*, fourth edition (*DSM-IV*) [22]. Thus, 332 women who met *DSM-IV* criteria for past or current MDD and 644 women with no history of MDD were enrolled in the HSMC. The remaining 3185 women from the target population were not enrolled in the study. For the purposes of the current study, women with any history of depression are referred to as “depressed” while those with no history of MDD are referred to as “non-depressed.” The Hamilton Rating Scale for Depression (HAM-D) [23] was also administered at baseline to measure depression symptom severity. Additional questionnaire items asked whether participants had ever used prescription antidepressants for a period of six months or more, how many depressive episodes they had experienced, age at the first depressive episode, and the total duration of all depressive episodes combined.

Details on sampling procedures and demographic comparisons of enrolled and non-enrolled women have been described previously [24, 25]. The HSMC was approved by the Institutional Review Board at Brigham and Women’s Hospital and all participants provided written informed consent.

Participants provided early follicular phase (day 2–4 of the menstrual cycle) blood specimens at study enrollment to measure reproductive hormones. For this analysis, we used baseline samples from a subset of women with and without a history of depression who were able to provide menstrually-timed blood specimens that were also used in a previous analysis of inhibin B [26].

### Anti-müllerian hormone (AMH) immunoassay

Serum samples were tested using the picoAMH kit (Ansh Labs, Webster, TX), with an assay sensitivity of 6 pg/ml, intra-assay CV < 6.5% and inter-assay CV < 11%. This assay is FDA approved and a clinically acceptable method of AMH measurement [27, 28]. Samples were analyzed for AMH in 401 women who provided menstrual-timed blood specimens at study enrollment and who were assessed in an earlier study of Inhibin-B that required multiple specimens [26]. The current analysis omitted participants who reported physician-diagnosed polycystic ovarian syndrome (PCOS) (*n* = 3), as this has been associated with increased AMH levels [29]. Participants with AMH levels ≥5.0 ng/mL (*n* = 29) were also omitted as this may be an indication of undiagnosed PCOS [30]. After these exclusions, 141 depressed women and 228 non-depressed women were available for analysis of baseline AMH levels. We assessed demographic and reproductive differences between women in whom we did and did not assess AMH levels. We found that women with and without AMH analyses were similar for most demographic and reproductive variables available. The exception was that a greater proportion of those with AMH analyzed were: a) depressed (43.7% vs 36.1%) and b) nulligravid (33.6% vs 16.2%). We address the impact of this limitation in the Discussion below.

### Demographic and reproductive characteristics

Self-reported data on demographics, reproductive health, medical history, and other lifestyle factors were collected during in-person interviews at baseline. Sociodemographic characteristics included age, race, education level and marital status. Body weight and height were used to calculate baseline body mass index (BMI, kg/m^2^), and participants were categorized into four BMI groups (underweight < 20, normal 20–24.9, overweight 25–29.9 and obese ≥30). Medical questionnaires elicited data on menstrual history, such as oral contraceptive use, prior pregnancies, number of livebirths, age at menarche, and average cycle length. Smoking status was assessed and categorized into groups (current/former and never-smoker) and pack-years (the number of packs of cigarettes smoked per day multiplied by the number of active smoking years) were calculated as a measure of smoking intensity. Select covariates were examined as potential effect measure modifiers, including age, parity, infertility history, current cycle regularity, smoking status, and BMI. Infertility history was defined as responding yes to the question “Did you ever try for more than 2 years to get pregnant or have problems carrying a pregnancy?” Current cycle regularity was defined as responding yes to the question “Are your cycles now generally regular, that is, usually predictable within 10 days?”

### Statistical analysis

Baseline AMH levels were dichotomized based on a clinically relevant threshold of 1.4 ng/mL. Diminished ovarian reserve is consistent with low AMH levels [31] defined as less than or equal to 1.4 ng/mL after accounting for use of the Ansh AMH assay method [27].

We used a modified Poisson regression model with a robust error variance [32] to estimate prevalence ratios (PR) and 95% confidence intervals (CI) for the association between depression history and AMH levels ≤1.4 ng/mL (i.e., diminished ovarian reserve). Age- and covariate-adjusted models were presented for depression and its characteristics. Potential confounders were identified from a review of the literature and the drawing of a directed acyclic graph. Covariates included in final multivariable models were age, BMI, smoking status, pack-years of smoking, parity, current menstrual cycle irregularity, age at menarche, and lifetime duration of oral contraceptive use. These variables were included in our adjusted models based on meeting criteria for confounding (> 10% difference in crude versus adjusted risk estimates) or because previous literature indicated their importance as confounders. In addition to categorical analyses, we used restricted cubic splines to model the association between selected variables (depression history, age, BMI, smoking) and AMH as a continuous outcome variable, without imposing linearity on the associations [33]. Each spline curve was fit with four knot points for AMH concentrations at the 20th, 40th, 60th, and 80th percentiles (0.100, 0.750, 1.750, 3.250 ng/nl).

Effect measure modification was assessed by stratifying on selected covariates that we hypothesized a priori might modify the association between depression and AMH. These variables included age, parity, smoking history, infertility history, and history of OC use. Associations between covariates and low AMH were also examined within each of the depressed and non-depressed cohorts. In secondary analyses, AMH levels were examined as a continuous outcome measure using restricted cubic splines to confirm model results. All analyses were performed in SAS v9.4 (SAS Institute, Cary, NC).

## Results

Although all women were between 36 and 45 years of age (Table 1), depressed women were slightly older than non-depressed women (23% vs. 13% were 44–45 years old, respectively). In addition, depressed women were more likely to have ever smoked cigarettes (55% depressed vs. 47% non-depressed) or to have used oral contraceptives (73% depressed vs. 67% non-depressed) in their lifetime. Depressed women were considerably more likely to be nulliparous (60% depressed vs. 37% non-depressed), but reported lower two-year infertility rates (11% depressed vs. 22% non-depressed). Body mass index (BMI), race, age at menarche, and average menstrual cycle lengths were similar between depressed and non-depressed women.

**Table 1:** Baseline health and sociodemographic characteristics of women with and without a history of depression, Harvard Study of Moods and Cycles, 1995–1998

	History of Depression (***n*** = 141)		No History of Depression (***n*** = 228)	
	n	%	n	%
Age, years				
36–37	17	12.1	36	15.8
38–39	28	19.9	48	21.1
40–41	38	27.0	65	28.5
42–43	26	18.4	50	21.9
44–45	32	22.7	29	12.7
Race/Ethnicity				
Non-Hispanic White	136	96.4	216	94.7
Black, Hispanic/Latina, Asian, Other	5	3.6	12	5.3
Education				
Less than college	32	22.7	71	31.1
College	41	29.1	77	33.8
Graduate School	68	48.2	80	35.1
Marital status				
Married	89	63.1	162	71.0
Single/Never married	27	19.2	48	21.1
Divorced/separated	25	17.7	18	7.9
Body mass index (BMI), kg/m^2^				
Underweight (< 20)	26	18.4	41	18.0
Normal weight (20-24.9)	61	43.3	97	42.5
Overweight (25-29.9)	35	24.8	57	25.0
Obese (≥30)	19	13.5	33	14.5
Smoking history				
Never smoker	64	45.4	121	53.1
Former smoker	54	38.3	78	34.2
Current smoker	23	16.3	29	12.7
Age at menarche, years				
≤ 11	27	19.2	36	15.8
12–14	85	60.3	141	61.8
≥ 15	29	20.6	51	22.4
Parity (livebirths)				
Nulliparous	84	59.6	85	37.3
1	23	16.3	33	14.5
≥ 2	34	24.1	110	48.3
Gravidity (pregnancies)				
Nulligravid	60	42.6	64	28.1
1–2	49	34.8	74	32.5
≥ 3	32	22.7	90	39.5
Oral contraceptive use, years				
Never	38	27.0	75	32.9
1	36	25.5	51	22.4
2–5	34	24.1	51	22.4
> 5	33	23.4	51	22.4
Menstrual cycle regularity^a^				
Irregular	8	5.7	21	9.2
Regular	133	94.3	207	90.8
Menstrual cycle length, days				
<26	24	17.0	36	15.8
26–29	85	60.3	142	62.3
≥ 30	32	22.7	50	21.9
History of infertility^b^	16	11.4	50	21.9
Ever used antidepressants^c^	88	62.4	3	1.3

a. Question asked “Are your cycles now generally regular, that is, usually predictable within 10 days?

b. Question asked “Did you ever try for more than 2 years to get pregnant or have problems carrying a pregnancy?”

c. Participants were asked if they had ever taken antidepressant medications for a period of 6 months of more

Overall, there was little association between depression history and baseline serum AMH levels (Table 2). The unadjusted prevalence of low AMH (≤1.4 ng/mL) was similar for depressed (57.5%) and non-depressed (57.9%) women, while depressed women had marginally higher serum levels of AMH (μ = 1.60 ng/mL, sd = 1.3) compared with non-depressed women (μ = 1.44 ng/mL, sd = 1.3). However, after adjusting for covariates, depression history showed a modest inverse association with low AMH (PR = 0.90, 95% CI: 0.75, 1.08), indicating a slightly lower likelihood of low ovarian reserve among depressed women.

**Table 2:** Association of depression history and severity characteristics with low levels of anti-müllerian hormone (≤1.4 ng/mL), Harvard Study of Moods and Cycles, 1995–1998

	AMH Level		Age-adjusted	Multivariable Ɨ	
	≤1.4 ng/mL				
	n	%	PR	Adjusted PR (95% CI)	
Model 1					
History of depression^a^					
No	132	57.9	1.00	1.00	Reference
Yes	81	57.5	0.92	0.90	(0.75, 1.08)
Model 2					
Antidepressant use					
Depressed, never used antidepressants	31	58.5	0.93	0.91	(0.71, 1.15)
Depressed, ever used antidepressants	50	56.8	0.92	0.89	(0.72, 1.11)
Model 3					
Depression & anxiety					
Depression only	41	56.2	0.91	0.89	(0.71, 1.12)
Depression and anxiety	40	58.8	0.94	0.91	(0.73, 1.13)
Model 4					
Hamilton Depression Rating Scale (HAM-D)^b^					
History of depression, HAM-D < 8	19	54.3	0.86	0.81	(0.59, 1.11)
History of depression, HAM-D ≥ 8	46	58.2	0.94	0.93	(0.75, 1.15)
Model 5					
Age of depression onset, years^c^					
< 25	42	59.2	1.03	0.97	(0.78, 1.20)
25–29	14	58.3	0.95	0.90	(0.64, 1.27)
≥ 30	22	53.7	0.83	0.81	(0.61, 1.09)
Model 6					
Number of depressive episodes^c^					
1–2	46	56.8	0.89	0.87	(0.71, 1.07)
≥ 3	32	58.2	0.98	0.97	(0.76, 1.24)
Model 7					
Total duration of depressive episodes, months^c^					
< 12	38	52.8	0.86	0.85	(0.68, 1.07)
12–35	16	61.5	1.01	1.00	(0.74, 1.36)
≥ 36	23	62.2	0.97	0.94	(0.72, 1.24)

Ɨ Models adjusted for age, age at menarche, cycle irregularity, parity, oral contraceptive use, smoking status at baseline, smoking pack-years, and body mass index (BMI). Analyses were restricted to those with AMH < 5.0 ng/mL, a potential indicator of elevated AMH due to polycystic ovary syndrome (PCOS)

a. Non-depressed serve as reference group for all exposure variables (displayed in first row only)

b. Hamilton Depression scores were missing for *n* = 27 (apx. 7% from both depressed and non-depressed cohort)

c. ‘Age of onset’ variable missing 5 responses; ‘number of episodes’ and ‘duration’ variables missing 6 responses

Results did not reveal a monotonic inverse association between greater depression severity and AMH levels. Associations for depressed women who had taken antidepressants (PR = 0.89, 95% CI: 0.72, 1.11) and those with comorbid anxiety at baseline (PR = 0.91, 95% CI: 0.73, 1.13) were similar to that of non-depressed women (Table 2). The association with low AMH was somewhat more pronounced for depressed than non-depressed women with lower symptom severity scores (HAM-D < 8) (PR = 0.81, 95% CI: 0.59, 1.11). Depressed women with later ages at first onset of depression (age ≥ 30) (PR = 0.81, 95% CI: 0.61, 1.09) and shorter total duration of depressive episodes (< 12 months) (PR = 0.85, 95% CI: 0.68, 1.07) were slightly more likely to show diminished ovarian reserve compared with non-depressed women, but the latter did not show evidence of a dose-response association.

After stratifying by age (Table 3), PRs for the association between depression and low AMH were 0.75 among women aged 36–40 years (95% CI: 0.52, 1.09) and 1.16 among women aged 41–45 years (95% CI: 0.95, 1.40). Similarly, PRs for the association between depression and low AMH were 0.77 among nulliparous women (95% CI: 0.59, 1.00) and 1.08 among parous women (95% CI: 0.85, 1.38). Results did not vary appreciably by menstrual cycle regularity (regular/irregular), smoking status (never/ever), or BMI (normal/overweight/obese).

**Table 3:** Association of depression history with low levels of anti-müllerian hormone (≤1.4 ng/mL), stratified by oral contraceptive use, parity, cycle regularity, smoking and body mass index (BMI), Harvard Study of Moods and Cycles, 1995–1998

	AMH Level (≤1.4 ng/mL)		Age-Adjusted	Multivariable Ɨ	
	n	%	PR^a^	Adjusted PR (95% CI)	
Age, years					
36–40	85	45.2	0.74	0.75	(0.52, 1.09)
41–45	128	70.7	1.11	1.16	(0.95, 1.40)
Parity (livebirths) ^c^					
Parous	117	58.5	1.07	1.08	(0.85, 1.38)
Nulliparous	96	56.8	0.78	0.77	(0.59, 1.00)
Infertility					
Fertile	178	58.8	0.96	0.98	(0.81, 1.18)
Infertile	35	53.0	0.58	0.65	(0.32, 1.33)
Cycle regularity ^b,c^					
Regular Cycle	191	56.2	0.97	0.96	(0.80, 1.16)
Irregular Cycle	22	75.9	0.75	0.70	(0.38, 1.29)
Baseline smoking status					
Ever-smokers	29	55.8	0.68	0.79	(0.50, 1.26)
Never-smokers	184	58.0	0.97	0.96	(0.80, 1.16)
Body mass index (BMI), kg/m^2^					
Normal weight (< 25)	128	56.9	0.87	0.85	(0.68, 1.08)
Overweight (25-29.9)	55	59.8	1.19	1.22	(0.85, 1.73)
Obese (≥30)	30	57.7	0.75	0.80	(0.48, 1.35)

Ɨ Covariates included age, age at menarche, irregular cycle, pack years smoking, and body mass index (BMI)

a. ‘Age-adjusted’ model reports crude prevalence ratios for stratified age-groups

b. Did not adjust for parity

c. Did not adjust for oral contraceptive use

**Fig. 1:**
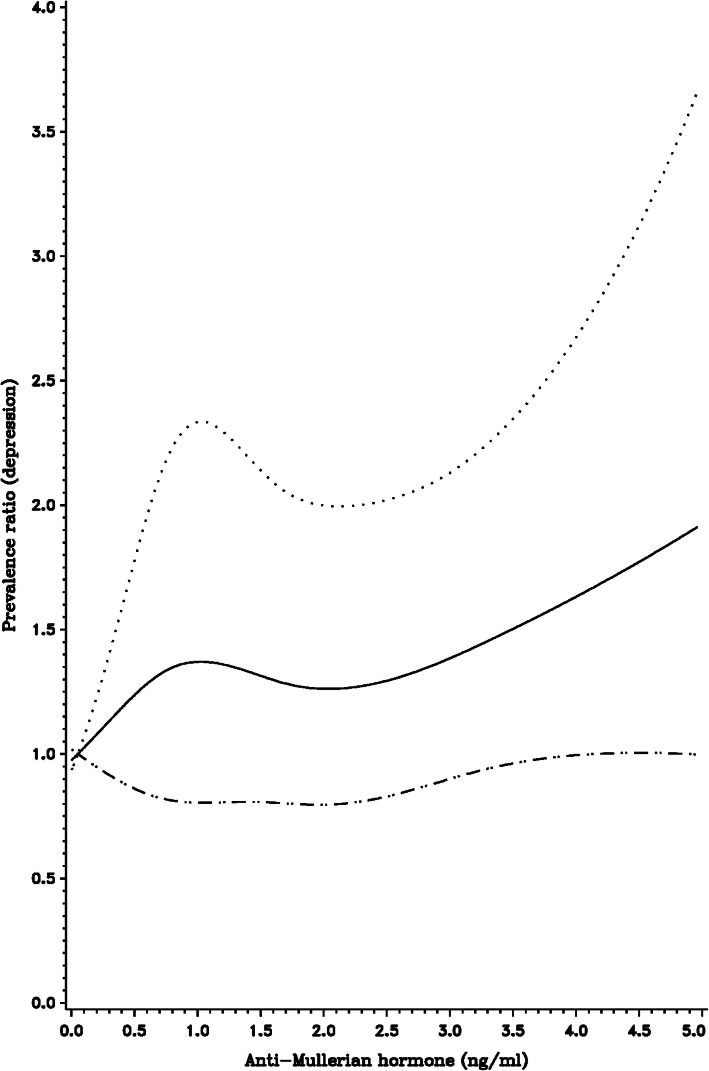
Restricted cubic spline displaying the association between depression history and AMH levels, with knot points at AMH levels of 0.100, 0.750, 1.750, 3.250. Adjusted for age, cycle irregularity, BMI, pack-years of smoking, age at menarche, and history of OC use

**Fig. 2:**
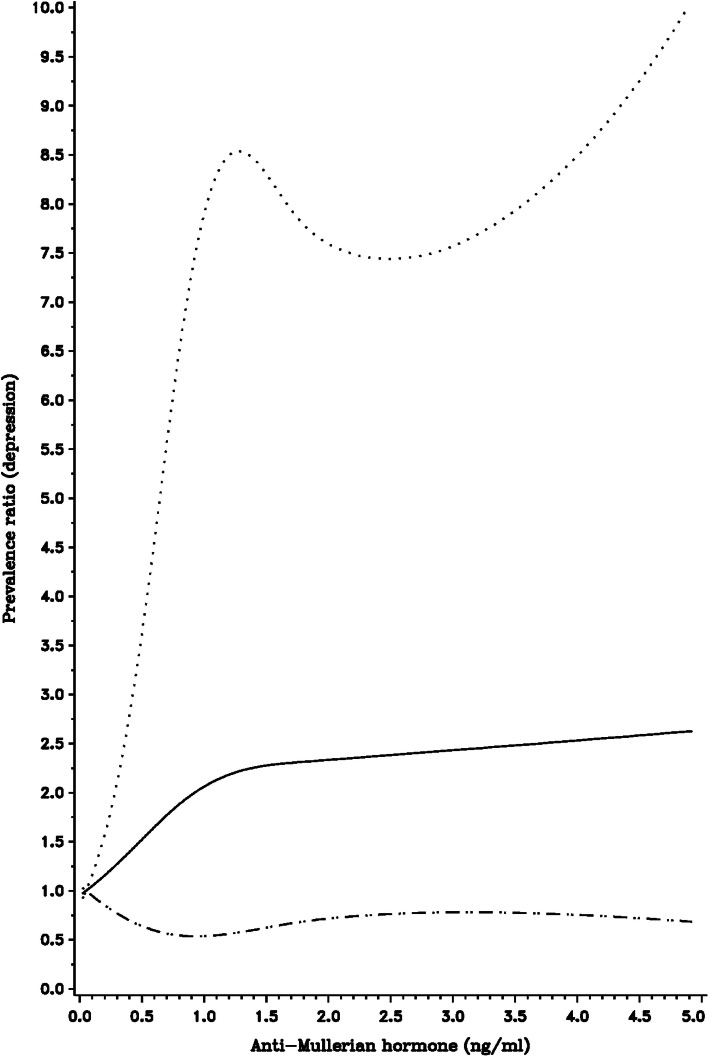
Restricted cubic spline displaying the association between depression history and AMH levels among women aged 35–39 years at enrollment, with knot points at AMH levels of 0.100, 0.750, 1.750, 3.250. Adjusted for cycle irregularity, BMI, pack-years of smoking, age at menarche, and history of OC use

**Fig. 3:**
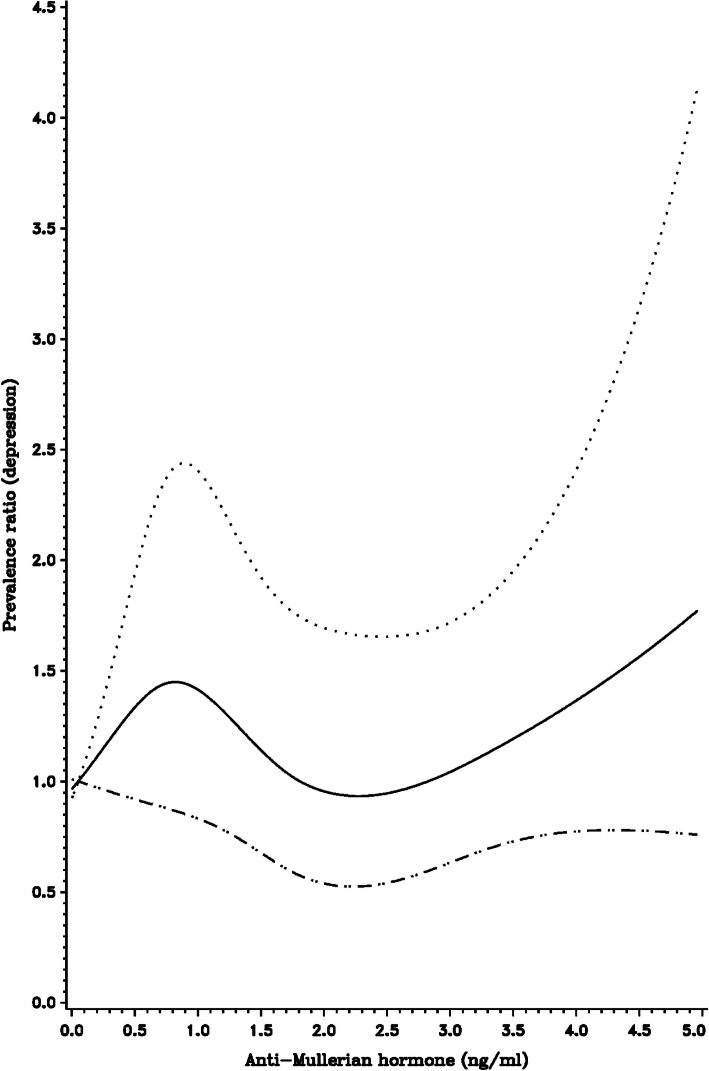
Restricted cubic spline displaying the association between depression history and AMH levels among women aged 40–44 years at enrollment, with knot points at AMH levels of 0.100, 0.750, 1.750, 3.250. Adjusted for cycle irregularity, BMI, pack-years of smoking, age at menarche, and history of OC use

Restricted cubic spline curves, in which AMH was modeled as a continuous variable, were generally consistent with the categorical analyses (Figs. 1, 2 and 3). Specifically, we observed a modest positive association between serum AMH levels and depression prevalence (Fig. 1), as well as the age-specific associations observed in previously-described stratified analyses (Figs. 2 and 3). Participant age was associated with lower AMH (Supplemental Fig. 1), as were pack-years of smoking (Supplemental Fig. 2). There was little association between BMI and AMH (Supplemental Fig. 3).

## Discussion

We hypothesized that women with a history of depression would show an earlier decrease in serum AMH levels, after controlling for age, based on prior studies suggesting that early perimenopause occurs to a greater extent among depressed women. However, our results do not support this hypothesis. Our data also showed no appreciable association between depression severity and serum AMH levels. Results for various depression characteristics (e.g., age at onset, duration of illness, number of episodes) also showed inconsistent associations with AMH concentrations. Finally, results based on a categorical cut point for “low” AMH concentrations (≤1.4 ng/ml) were similar to those based on AMH concentrations as a continuous variable.

Prior studies consistently describe an age-related decline in serum AMH concentrations [34–37], as well as modest reductions in serum AMH concentrations related to cigarette smoking [6, 38]. BMI has been reported to have an inverse relationship with serum AMH in some [39–42], but not all [6, 43, 44], studies. In the present study, AMH concentrations tended to decrease with advancing age and greater intensity of cigarette smoking, but little association was observed for BMI. Women with a history of depression were slightly older and were more frequently smokers, yet, analysis of AMH concentrations with adjustment for covariates did not appreciably alter results.

A prior study of the relationship between depressive symptoms (as measured by the Beck Depression Inventory) and AMH concentrations [45] in late-reproductive aged women corroborates our findings. The authors reported similar AMH levels regardless of depression. However, their conclusion was based on results from a bivariate analysis that did not adjust for age and other covariates. It is noteworthy that psychological stress, a different but related neuropsychological phenomenon, was associated with lower AMH concentrations among a large group of infertile women [46].

We previously examined the association between depression and inhibin B in the present subcohort [26]. In that analysis, inhibin B was not markedly different between the depressed and non-depressed women at baseline or during 18 months of follow-up, and showed a slight inverse association. Inhibin B increases during the follicular phase of the menstrual cycle and peaks with the formation of the dominant follicle mid-cycle. This follicle growth reflects a gonadotropin-dependent process. Although ovarian granulosa cells are the source of both inhibin B and AMH, the roles and secretion of these hormones are quite different. Unlike inhibin B, AMH is a product of the small pre-antral follicles and is independent of FSH secretion. AMH is a direct measure of the follicles in reserve in the ovary, with concentrations highly correlated with chronological age and ultrasound-determined antral follicle counts. AMH is superior to inhibin B in predicting ovarian aging and menopause [47]. One might speculate that either ovarian reserve or ovarian development is altered by depression, but collectively these data suggest neither is true. Although, in this same dataset, we previously showed an association between depression and earlier onset of perimenopause based on menstrual cycle changes [20].

Depression may, on the other hand, influence the reproductive axis, perhaps primarily at the level of the pituitary gland and/or hypothalamus. In the original HSMC study of 976 women, 332 with a history of depression at baseline had an earlier onset of perimenopause during 3 years of follow-up, with perimenopause being defined as changes in menstrual cycle regularity and bleeding patterns. These women also exhibited higher serum follicle stimulating and luteinizing hormone concentrations, and lower estradiol concentrations, than those without depression. The mechanism by which depression might influence these hormones is unknown; however, combined with the lack of altered ovarian inhibin B and AMH concentrations, neuroendocrine candidates are likely mediators. The effect of depression on hypothalamic factors that control pituitary gonadotropin secretion, such as GnRH and kisspeptin, may be worthwhile to investigate.

Our data were limited by the fact that only a subset (*n* = 369) of samples from the larger study group were assayed for AMH, and a greater proportion of depressed women were included in the subcohort relative to the complete HSMC cohort. If selection into the AMH substudy also depended on ovarian reserve (e.g., the availability of menstrually-timed blood specimens due to a lower likelihood of amenorrhea/irregular cycling), then the observed positive association between depression and AMH could be spurious. Likewise, AMH concentrations were assayed in a greater proportion of nulligravid depressed women than nulliparous non-depressed women. If nulligravidity is associated with greater ovarian reserve, this could explain the observed better ovarian reserve among depressed women. This is not likely to be an artifact of age since we did not see any major differences in specimen availability by age.

Laboratory development of AMH immunoassays has changed rapidly in recent years and continues to undergo improvement. The original methods were manual and laborious to perform. Assay protocols were modified as interfering substances were identified. Newer methods now include automation. Nevertheless, we still lack a universal assay calibration which results in different absolute values obtained by each method [27, 28]. Investigators are responsible for knowing which assay is being used and the appropriate reference ranges for that method. Our results were obtained using the most sensitive assay available (Ansh picoAMH), with a lower limit of 6 pg/mL. This is ideal for women of late reproductive age, where AMH levels are expected to be relatively low, and beyond the lower limit of detection (~ 100 pg/mL) in other assay methods. The present assay results demonstrate the expected age-related decline in serum AMH levels. In fact, the distribution of AMH concentrations by age (Supplemental Fig. 1) is similar to that observed by Fong et al. who used controls from previous studies with a wide range of timing between assay and data collection, and whose specimens had been thawed and refrozen [48]. In addition, although these specimens had been frozen for several years prior to analysis, any effect of storage would similarly impact samples from depressed and non-depressed women.

A limitation of the current study is the cross-sectional nature of the data. In addition, the HSMC sample over-represents non-Hispanic white women, which precluded the possibility of examining differences in the associations among women of different racial and ethnic groups.

## Conclusions

In summary, our findings do not support a deleterious effect of depression on ovarian reserve as measured by AMH. These findings are consistent with our earlier assessment of inhibin B and depression.

## Supplementary information

**Additional file 1: Figure S1.** Restricted cubic spline displaying the association between age and AMH levels, with knot points at AMH levels of 0.100, 0.750, 1.750, 3.250. Adjusted for BMI and pack-years of smoking. **Figure S2.** Restricted cubic spline displaying the association between pack-years of smoking and AMH levels, with knot points at AMH levels of 0.100, 0.750, 1.750, 3.250. Adjusted for age and BMI. **Figure S3.** Restricted cubic spline displaying the association between BMI and AMH levels, with knot points at AMH levels of 0.100, 0.750, 1.750, 3.250. Adjusted for age and pack-years of smoking.

## Data Availability

The datasets used and/or analyzed during the current study are available from the corresponding author on reasonable request.

## Abbreviations

AMH: Anti-müllerian hormone
BMI: Body mass index
CES-D: Center for Epidemiologic Studies 20-item depression symptoms inventory
CI: Confidence interval
DSM-IV: Diagnostic and Statistical Manual of Mental Disorders, fourth edition
HAM-D: Hamilton Rating Scale for Depression
HSMC: Harvard Study of Moods and Cycles
MDD: Major depressive disorder
OC: Oral contraceptives
PCOS: Polycystic ovarian syndrome
PR: Prevalence Ratios
SCID: Structured clinical interviews for depression
