# Supplementary material for: Association between a history of depression and anti-müllerian hormone among late-reproductive aged women: the Harvard study of moods and cycles
Source: Womens Midlife Health. 2020 Sep 1;6:9. doi: 10.1186/s40695-020-00056-x (PMC7461252; doi:10.1186/s40695-020-00056-x)
Supplement: Supplementary file 1 — Additional file 1: Figure S1. Restricted cubic spline displaying the association between age and AMH levels, with knot points at AMH levels of 0.100, 0.750, 1.750, 3.250. Adjusted for BMI and pack-years of smoking. Figure S2. Restricted cubic spline displaying the association between pack-years of smoking and AMH levels, with knot points at AMH levels of 0.100, 0.750, 1.750, 3.250. Adjusted for age and BMI. Figure S3. Restricted cubic spline displaying the association between BMI and AMH levels, with knot points at AMH levels of 0.100, 0.750, 1.750, 3.250. Adjusted for age and pack-years of smoking. [file 40695_2020_56_MOESM1_ESM.docx]

**Supplemental Figure 1.** *Restricted cubic spline displaying the association between age and AMH levels, with knot points at AMH levels of 0.100, 0.750, 1.750, 3.250. Adjusted for BMI and pack-years of smoking.*

*
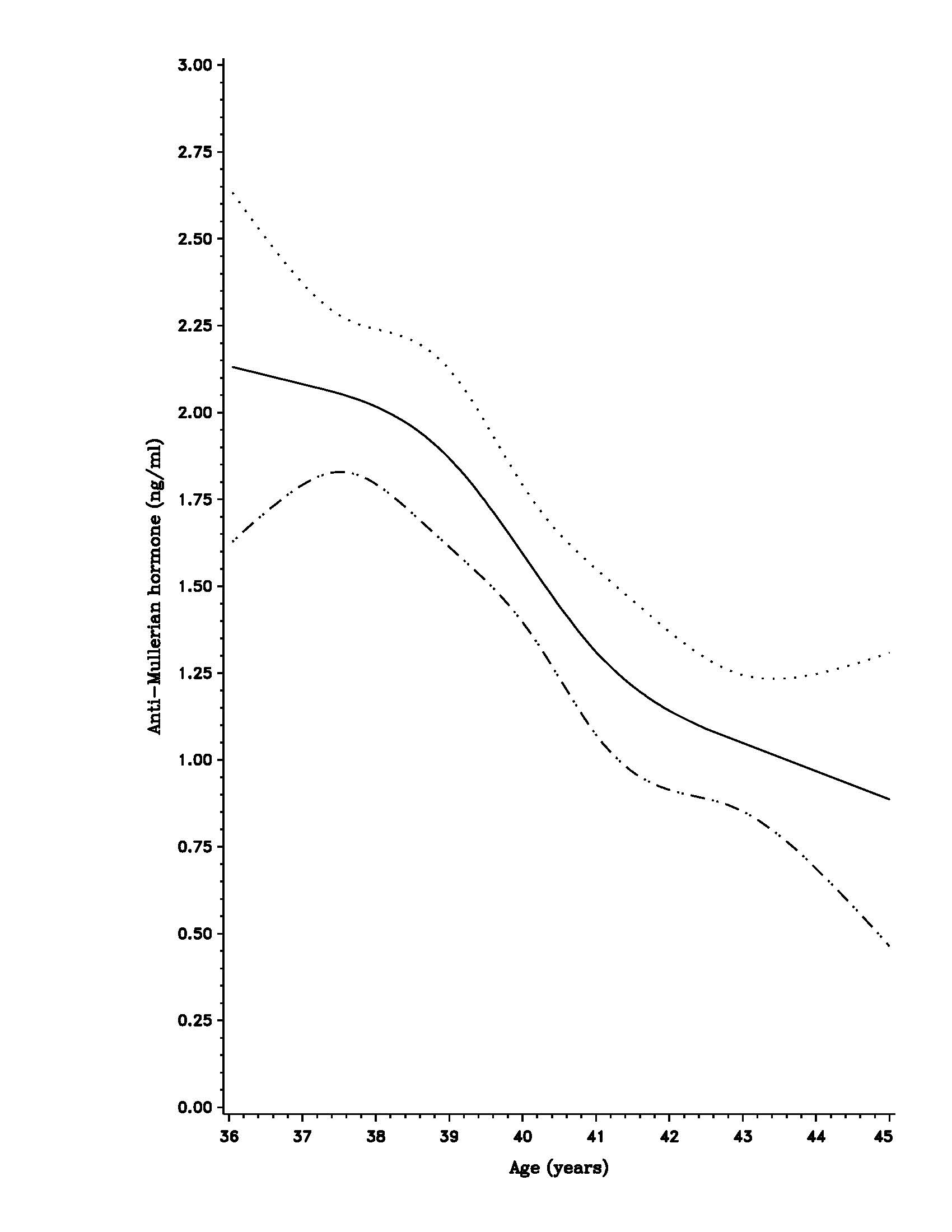
*

**Supplemental Figure 2.** *Restricted cubic spline displaying the association between pack-years of smoking and AMH levels, with knot points at AMH levels of 0.100, 0.750, 1.750, 3.250. Adjusted for age and BMI.*

*
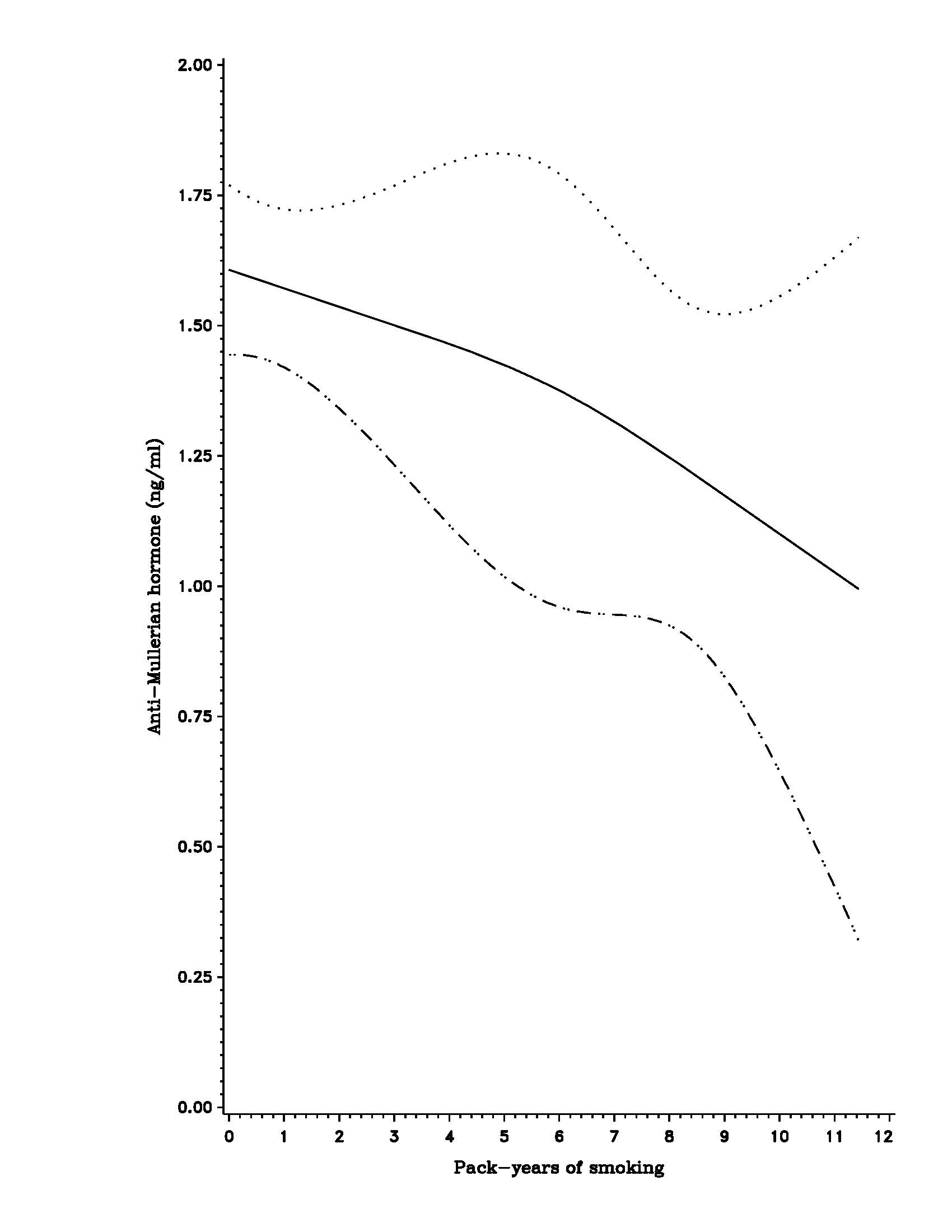
*

**Supplemental Figure 3.** *Restricted cubic spline displaying the association between BMI and AMH levels, with knot points at AMH levels of 0.100, 0.750, 1.750, 3.250. Adjusted for age and pack-years of smoking.*

*
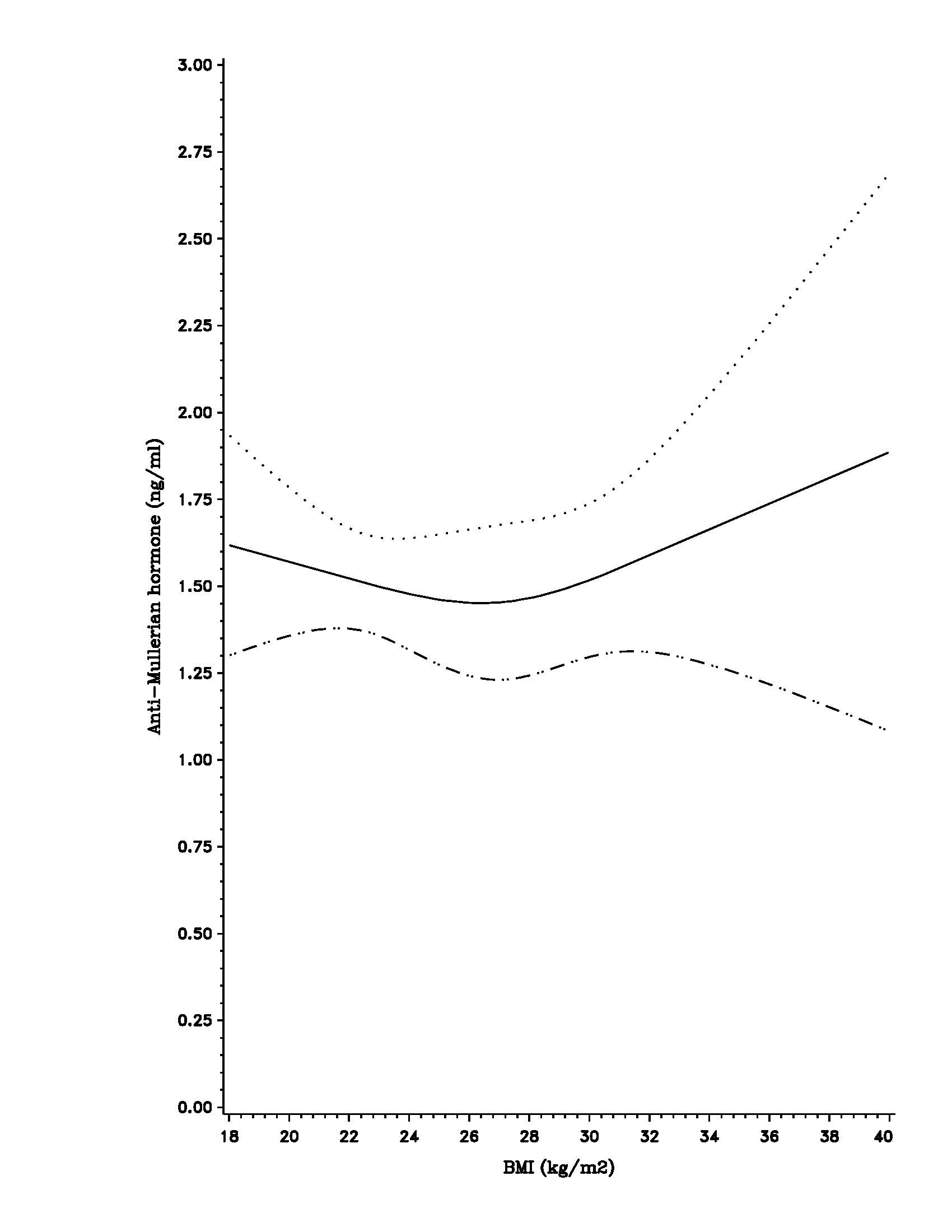
*
